# Supplementary material for: MVMRmode: Introducing an R package for plurality valid estimators for multivariable Mendelian randomisation
Source: PLoS One. 2024 May 7;19(5):e0291183. doi: 10.1371/journal.pone.0291183 (PMC11075861; doi:10.1371/journal.pone.0291183)
Supplement: S1 Table — (DOCX) [file pone.0291183.s001.docx]

|  | | | No bias | 10% balanced pleiotropic | 20% balanced pleiotropic | 40% balanced pleiotropic | 10% directional pleiotropic | 20% directional pleiotropic | 40% directional pleiotropic |
| --- | --- | --- | --- | --- | --- | --- | --- | --- | --- |
| SD | IVW | Exposure 1 | 0.025 | 0.084 | 0.12 | 0.167 | 0.084 | 0.123 | 0.171 |
|  |  | Exposure 2 | 0.025 | 0.087 | 0.118 | 0.165 | 0.085 | 0.122 | 0.17 |
|  | MR Egger | Exposure 1 | 0.089 | 0.139 | 0.186 | 0.249 | 0.144 | 0.186 | 0.244 |
|  |  | Exposure 2 | 0.049 | 0.125 | 0.167 | 0.216 | 0.125 | 0.169 | 0.229 |
|  | Median | Exposure 1 | 0.031 | 0.035 | 0.041 | 0.05 | 0.035 | 0.039 | 0.051 |
|  |  | Exposure 2 | 0.032 | 0.037 | 0.041 | 0.05 | 0.036 | 0.039 | 0.052 |
|  | multivariable-CM | Exposure 1 | 0.028 | 0.03 | 0.033 | 0.044 | 0.031 | 0.042 | 0.108 |
|  |  | Exposure 2 | 0.028 | 0.03 | 0.034 | 0.044 | 0.03 | 0.039 | 0.103 |
|  | multivariable-MBE | Exposure 1 | 1.026 | 2.126 | 1.813 | 5.014 | 3.448 | 3.507 | 9.546 |
|  |  | Exposure 2 | 3.708 | 2.04 | 2.882 | 11.9 | 3.704 | 3.413 | 4.021 |
| Coverage | IVW | Exposure 1 | 0.948 | 0.946 | 0.945 | 0.951 | 0.945 | 0.932 | 0.904 |
|  |  | Exposure 2 | 0.941 | 0.938 | 0.942 | 0.944 | 0.934 | 0.908 | 0.878 |
|  | MR Egger | Exposure 1 | 0.639 | 0.873 | 0.892 | 0.918 | 0.873 | 0.904 | 0.938 |
|  |  | Exposure 2 | 0.828 | 0.924 | 0.943 | 0.958 | 0.925 | 0.935 | 0.945 |
|  | Median | Exposure 1 | 0.974 | 0.964 | 0.94 | 0.926 | 0.962 | 0.956 | 0.908 |
|  |  | Exposure 2 | 0.963 | 0.949 | 0.926 | 0.921 | 0.939 | 0.924 | 0.873 |
|  | multivariable-CM | Exposure 1 | 0.909 | 0.916 | 0.923 | 0.907 | 0.919 | 0.884 | 0.692 |
|  |  | Exposure 2 | 0.894 | 0.904 | 0.91 | 0.903 | 0.921 | 0.925 | 0.739 |
|  | multivariable-MBE | Exposure 1 | 0.995 | 0.994 | 0.996 | 0.995 | 0.991 | 0.993 | 0.977 |
|  |  | Exposure 2 | 0.991 | 0.991 | 0.995 | 0.992 | 0.992 | 0.996 | 0.986 |

S1 Table: Results for additional outcomes when both exposures cause the outcome, and exposure 2 is pleiotropic.
