# Supplementary material for: MVMRmode: Introducing an R package for plurality valid estimators for multivariable Mendelian randomisation
Source: PLoS One. 2024 May 7;19(5):e0291183. doi: 10.1371/journal.pone.0291183 (PMC11075861; doi:10.1371/journal.pone.0291183)
Supplement: S2 Table — (DOCX) [file pone.0291183.s002.docx]

|  | | | No bias | 20% balanced pleiotropic | 40% balanced pleiotropic | 60% balanced pleiotropic | 10% directional pleiotropic | 20% directional pleiotropic | 40% directional pleiotropic |
| --- | --- | --- | --- | --- | --- | --- | --- | --- | --- |
| SD | IVW | Exposure 1 | 0.008 | 0.079 | 0.117 | 0.167 | 0.09 | 0.118 | 0.173 |
|  |  | Exposure 2 | 0.008 | 0.079 | 0.116 | 0.166 | 0.091 | 0.118 | 0.167 |
|  | MR Egger | Exposure 1 | 0.011 | 0.111 | 0.162 | 0.23 | 0.123 | 0.163 | 0.232 |
|  |  | Exposure 2 | 0.011 | 0.109 | 0.158 | 0.217 | 0.12 | 0.158 | 0.229 |
|  | Median | Exposure 1 | 0.01 | 0.012 | 0.014 | 0.02 | 0.012 | 0.014 | 0.021 |
|  |  | Exposure 2 | 0.01 | 0.012 | 0.014 | 0.021 | 0.012 | 0.014 | 0.021 |
|  | multivariable-CM | Exposure 1 | 0.008 | 0.012 | 0.021 | 0.043 | 0.024 | 0.064 | 0.134 |
|  |  | Exposure 2 | 0.008 | 0.013 | 0.021 | 0.045 | 0.024 | 0.064 | 0.143 |
|  | multivariable-MBE | Exposure 1 | 0.268 | 1.379 | 4.344 | 7.928 | 1.974 | 4.892 | 3.578 |
|  |  | Exposure 2 | 0.411 | 2.459 | 2.76 | 16.764 | 1.667 | 2.962 | 16.847 |
| Coverage | IVW | Exposure 1 | 0.957 | 0.956 | 0.947 | 0.957 | 0.935 | 0.931 | 0.902 |
|  |  | Exposure 2 | 0.946 | 0.962 | 0.951 | 0.945 | 0.922 | 0.92 | 0.883 |
|  | MR Egger | Exposure 1 | 0.948 | 0.953 | 0.945 | 0.954 | 0.94 | 0.963 | 0.95 |
|  |  | Exposure 2 | 0.951 | 0.949 | 0.944 | 0.947 | 0.944 | 0.958 | 0.948 |
|  | Median | Exposure 1 | 0.971 | 0.98 | 0.957 | 0.919 | 0.968 | 0.965 | 0.891 |
|  |  | Exposure 2 | 0.972 | 0.963 | 0.954 | 0.911 | 0.972 | 0.959 | 0.907 |
|  | multivariable-CM | Exposure 1 | 0.954 | 0.953 | 0.921 | 0.861 | 0.897 | 0.633 | 0.235 |
|  |  | Exposure 2 | 0.947 | 0.951 | 0.917 | 0.854 | 0.881 | 0.65 | 0.247 |
|  | multivariable-MBE | Exposure 1 | 0.994 | 0.992 | 0.992 | 0.99 | 0.992 | 0.987 | 0.989 |
|  |  | Exposure 2 | 0.997 | 0.995 | 0.99 | 0.989 | 0.991 | 0.99 | 0.98 |

S2 Table: Results for additional outcomes when neither exposure cause the outcome, and exposure 2 is pleiotropic.
