# Supplementary material for: MVMRmode: Introducing an R package for plurality valid estimators for multivariable Mendelian randomisation
Source: PLoS One. 2024 May 7;19(5):e0291183. doi: 10.1371/journal.pone.0291183 (PMC11075861; doi:10.1371/journal.pone.0291183)
Supplement: Attachment — Submitted filename: r2 response letter v2.docx [file pone.0291183.s003.docx]

Suyan Tian

Academic Editor

PLOS ONE

Dear Dr Tian,

We would like to thank you and the reviewer for again taking the time to assess our article "MVMRmode: Introducing an R package for plurality valid estimators for multivariable Mendelian randomisation”, and providing such generous feedback. I apologise for most of these errors being typos – I’m badly dyslexic and struggle to pick up this type of mistake. Please find below our point-by-point response to the comments bellow where we have implemented all of the suggested changes.

Yours,

Benjamin Woolf

Journal Requirements:

“Please review your reference list to ensure that it is complete and correct. If you have cited papers that have been retracted, please include the rationale for doing so in the manuscript text, or remove these references and replace them with relevant current references. Any changes to the reference list should be mentioned in the rebuttal letter that accompanies your revised manuscript. If you need to cite a retracted article, indicate the article’s retracted status in the References list and also include a citation and full reference for the retraction notice.”

To the best of our knowledge we have not cited any retracted papers. In the process of revising the manuscript, it has come to our attention that other plurality-valid estimators for multivariable Mendelian randomization (MVMR) have been published (either as a journal article or as a pre-print). We have amended the Introduction and Discussion to clarify that this is no longer the first plurality-valid MVMR method, and added reference to these methods.

**Reviewers' comments**

**Reviewer #1:**

1. “a small typo (intuitive (adjective) should be replaced by "intuition" or "intuitive way")”

Thank you for picking this up, we have changed “intuitive” to " intuitive way "

**Reviewer #2:** 

1. “There are still some typos and unclear parts you might want to correct, for example: (i) the 2,, in the sentence “Now suppose we have estimates for two exposures, denoted by 1 and 2. 1, and 2,,” right after equation (1).”

Thank you for picking this up, I have removed the second comma form $\beta_{x_{2},i}$

2. “(ii) In simulation part, it’s mentioned that 200 SNPs are generated and generation of E1, E_PI includes the first 50 and first 100 SNPs, respectively. Do you use all the 200 SNPs or only the first 100 ones? Also there is a small p in the definition of O_N;P, I think you mean p = P / 100 * 200, is that correct?”

Thank you very much for picking this up. After double checking with the code, it should be 200 SNPs for both. I’m sorry for not having picked this up this version control issue before. I have changed the ‘P’ to a ‘p’.

3. “(iii) In the definition of O_E1, EPI;P, I think the 100 should be P or p?”

Thank you again for picking this up, it should be ‘p’. I have changed the ‘100’ to ‘p’

4. “(iv) ‘causals’ should be causal in the discussion of coverage as the additional outcomes in the Result section for simulation.”

Thank you for picking these up, we have made the suggested changes.

5. “Will you conclude that multivariable-MBE is not that useful in practice? Or provide some suggestions for under what circumstance this method might work? Maybe an inclusion of the MV-MBE performance under different levels of pleiotropy can be used to better explain why it fail in this framework, or include the sd for the estimator to support the argument “probably due to the greater uncertainty in the estimates”.

We agree with the reviewer, and have clarified this in the text:

Multivariable-MBE was sufficiently imprecise that it is likely to be uninformative in practice, and we would therefore suggest that, when needed, researchers use another robust multivariable method instead.

The claim “probably due to the greater uncertainty in the estimates” relates to the univariable mode-based method, which has been shown to have variable estimates in previous published comparisons of univariable Mendelian randomization methods. We now provide a citation for this claim.

6. “Just for comprehensive analysis, a more detailed discussion for more than two exposures will be ideal I think, will complicate the analysis for sure though. Because I’m kind of curious how different methods in this framework affect the final results and this kind of analysis, e.g. some patterns shown in the simulation, might provide some insights for your framework as well.”

We agree with the reviewer that this is a limitation of the current manuscript. However, due to the limited performance of the proposed methods in the current simulation scenarios, and the recent publication of other plurality-robust methods, we did not this it would be interesting to readers to provide a comparison of these methods in a more complex scenario.

In the text we have added the following:

“A further limitation of this work is that we have only considered the scenario with two exposures in our simulation study. However, the framework we introduce in this paper does naturally extend to consider more than two exposures by using multivariable IVW in the first stage.”
